# An Evolutionarily Conserved Mechanism for Activity-Dependent Visual Circuit Development

**DOI:** 10.3389/fncir.2016.00079

**Published:** 2016-10-21

**Authors:** Kara G. Pratt, Masaki Hiramoto, Hollis T. Cline

**Affiliations:** ^1^Program in Neuroscience, Department of Zoology and Physiology, University of WyomingLaramie, WY, USA; ^2^Department of Molecular and Cellular Neuroscience and The Dorris Neuroscience Center, The Scripps Research InstituteLa Jolla, CA, USA

**Keywords:** visual system plasticity, retinal waves, Hebb

## Abstract

Neural circuit development is an activity-dependent process. This activity can be spontaneous, such as the retinal waves that course across the mammalian embryonic retina, or it can be sensory-driven, such as the activation of retinal ganglion cells (RGCs) by visual stimuli. Whichever the source, neural activity provides essential instruction to the developing circuit. Indeed, experimentally altering activity has been shown to impact circuit development and function in many different ways and in many different model systems. In this review, we contemplate the idea that retinal waves in amniotes, the animals that develop either in ovo or utero (namely reptiles, birds and mammals) could be an evolutionary adaptation to life on land, and that the anamniotes, animals whose development is entirely external (namely the aquatic amphibians and fish), do not display retinal waves, most likely because they simply don’t need them. We then review what is known about the function of both retinal waves and visual stimuli on their respective downstream targets, and predict that the experience-dependent development of the tadpole visual system is a blueprint of what will be found in future studies of the effects of spontaneous retinal waves on instructing development of retinorecipient targets such as the superior colliculus (SC) and the lateral geniculate nucleus.

## Introduction

Spontaneous neural activity, defined here as self-generated electrical activity that is not driven by afferent input, exists in many amniote sensory systems during their development. This activity provides important instructions for circuit development and maturation. For example, spontaneous activity of the cochlear inner hair cells promotes the maturation of central auditory pathways before hearing onset in mammals (Wang and Bergles, 2015), spontaneous firing in olfactory sensory neurons is required for the formation of the olfactory sensory map (Yu et al., 2004; Lorenzon et al., 2015), and spontaneous retinal waves in the developing visual system, prior to visual experience, drive topographic map formation in downstream targets such as the superior colliculus (SC) and lateral geniculate nucleus (Torborg and Feller, 2005). In amphibian larvae, whose development is completely external, visual stimuli, instead of spontaneous retinal waves, drives retinal ganglion cells (RGCs) and this activity is known to instruct many aspects of development of this circuit (Sin et al., 2002; Ruthazer et al., 2003; Dong et al., 2009; Xu et al., 2011; Udin, 2012; Hiramoto and Cline, 2014). In this review, we discuss the role of activity in the development of topographic maps, neuronal structure and function and the maturation of neuronal circuits in the developing visual system. We first focus on the role of spontaneous retinal waves in amniotes, how they could be an evolutionary adaptation to developing on dry land in eggs or *in utero*, and recent findings about the consequence of these waves on their downstream targets. Next, we discuss the development of the amphibian visual system, and how the instructional activity in RGCs is generated by visual stimuli from the environment rather than retinal waves. We provide a comprehensive summary of the consequences of visual experience on the development of this circuit, underscoring both the importance of neural activity in circuit development and the advantages of the tadpole model for the study of circuit development. Lastly, we mention striking similarities between activity-dependent processes in the amphibian retinotectal circuit and those in non-sensory regions of the developing mammalian brain, suggesting that the fundamental mechanisms by which visual activity drives circuit development in tadpoles are conserved throughout the CNS of many species.

### Retinal Waves are Expressed in Amniotes But not in Non-Amniotes

While spontaneous retinal waves have been well described and studied in embryonic retinas of amniotes such as turtles (Sernagor and Grzywacz, 1996, 1999), chicks (Wong et al., 1998), ferrets (Meister et al., 1991; Wong et al., 1993), rodents (Torborg and Feller, 2005; Ackman et al., 2012) and primates (Warland et al., 2006), this spontaneous patterned activity is not present in the retina of amphibians (Demas et al., 2012) nor fish (Kolls and Meyer, 2002). This dichotomy suggests the intriguing possibility that retinal waves are an evolutionary adaptation in response to the transition from life in the water to life on the land, when the transparent jelly coat of the aquatic anamniote embryo was replaced by a hard opaque shell for birds and reptiles, or a uterus for mammals. Developing in ovo or *in utero* is well-suited for survival on dry land, but these protective environments keep the embryo literally in the dark, devoid of visual stimuli during periods of brain development when neurons are extending processes and establishing nascent connections, and when circuit connectivity is being refined. In contrast, the development of aquatic amphibians and fish, from fertilization onwards, takes place externally, with embryos surrounded by nothing more than a transparent coat of jelly and larvae being exposed to complex sensory environments. This means that these anamniote embryos and larvae are always exposed to the external visual scene. Natural visual stimulation of the photoreceptors and retinal interneurons activates RGCs and transmits activity to retinal axons in targets as soon as synapses are formed (Holt and Harris, 1983). Consequently, spontaneous retinal waves are not needed to activate RGCs and convey patterned activity to the central retinal targets in anamniotes. In fact, one might anticipate that retinal waves in the presence of natural visual stimuli would likely interfere with important instructional information provided by environmental visual cues.

**Figure 1:**
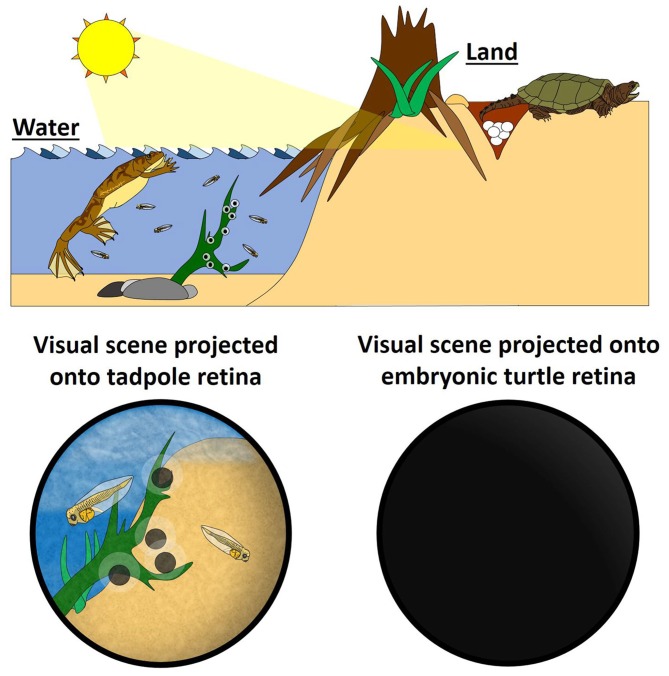
**Retinal waves could be an evolutionary adaptation for visual systems developing in the absence of visual stimuli.** (Left) In the water, fertilization of fish and aquatic amphibians takes place externally, so the embryos develop with the benefit of direct sensory stimuli. This natural visual stimulation activates retinal ganglion cells (RGCs), providing the activity for activity-dependent mechanisms that instruct the formation and maturation of the visual system. Retinal waves are not needed to generate RGC activity in anamniotes, and, consistent with this, the anamniote retina does not express retinal waves. (Right) On land, development of amniotes—by definition—takes place *in utero* or in ovo, and so these embryos do not experience natural visual scenes. RGCs still get activated, however, by self-generating spontaneous waves of activity *(Schematic by Harley Yerdon)*.

We suggest that, for amniotes, retinal waves could be an evolutionary adaptation to developing in the absence of patterned visual stimulation and serve as a proxy for visual experience (natural vision) in anamniotes (Figure 1). If this were true, it would be expected that retinal waves and visual experience would share common functions in the context of visual system development. In fact, emerging evidence indicates parallels between the role of visual stimulation in anamniotes and retinal waves in amniotes.

### The Function of Retinal Waves in Amniotes

In the absence of external visual stimuli, amniotes are born or hatch with an impressive amount of their visual system already wired and capable of detecting and processing visual information. Although earlier *in vitro* and *in vivo* electrophysiological studies revealed that RGCs are spontaneously active (Mastronarde, 1983; Galli and Maffei, 1988) it was not until rather recently that bona fide waves have been recorded *in vivo* using calcium imaging (Ackman et al., 2012). The ability to visualize retinal waves *in vivo* makes it possible to address, directly, fundamental questions about the function of retinal waves, in particular, how these waves may contribute to developmental events in the RGC targets. Ackman et al. (2012) imaged retinal waves in mice *in vivo*, from the RGC somata to their axon terminals in the SC, and found that the spontaneous retinal waves drive the same spatiotemporal pattern of wave activity in their postsynaptic SC targets. In other words, the waves in the postsynaptic SC neurons match the RGC waves in space and time. This suggests that patterned spontaneous activity generated in the retina provides a template of patterned activity that could instruct the development of higher-order circuits in the visual system (Ackman et al., 2012). This study also demonstrated that retinal waves have defined—not random—initiation sites: retinal waves are initiated in the ventro-temporal retina, and they tend to propagate toward dorso-nasal retina. Similarly, waves in the retinal axons within SC initiate in the rostral-medial region of the SC and propagate to the caudal-lateral region, indicative of the topographic organization of the retinocollicular projection that forms based on instructive signals from spontaneous retinal waves prior to vision in amniotes (Torborg and Feller, 2005). In addition, by imaging calcium transients in SC neurons, it is clear that waves of retinal activity drive postsynaptic collicular activity. Ackman et al. (2012) interpret these data as a way in which retinal waves contribute to the development of direction selectivity in collicular neurons as well as higher order neurons in cortex.

These studies demonstrate that retinal waves expressed in amniotes are essential for supplying the specific temporal and spatial patterns of activity to the RGCs and thereby, via correlation-based mechanisms, guiding RGC inputs to precise postsynaptic targets. Likewise, in the anamniote, visual experience supplies a similar type of patterned activation of RGCs such that neighboring RGCs are most correlated and the further apart they are, the less they are correlated. The similar roles of retinal waves in amniotes and visual stimuli in anamniotes are highlighted in the next section as we describe the role of visual stimuli in the developing visual system of the *Xenopus* tadpole.

### Function of Vision in the Amphibian Embryo

**Figure 2:**
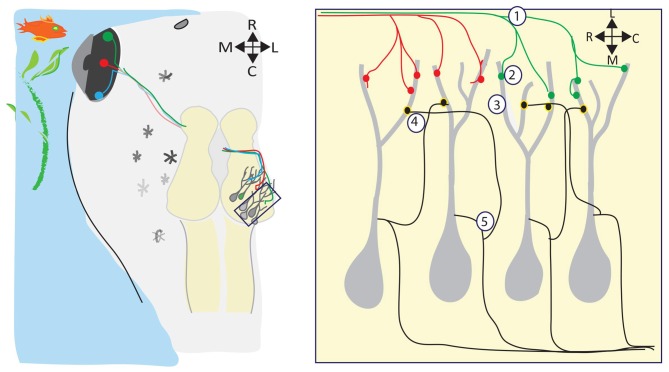
**Visual experience regulates many aspects of retinotectal circuit development.** (Left) The retinotectal circuit is comprised of the RGCs in the eye which project their axons to the optic tectum, the primary visual processing area in tadpoles and frogs. Retinotectal axons extend in the optic tract across the ventral midline of the brain, and then extend dorsally and caudally to their final destination, the contralateral optic tectum. Here, RGC axons branch out and form synapses with postsynaptic tectal neurons, forming a topographic map of visual space within the tectal neuropil such that near-neighbor RGCs synapse with near-neighbor tectal neurons. Visual stimuli in the tadpole’s environment activate the RGCs. During retinotectal circuit development, this visually-driven activity refines the retinotopic map (1), drives the development and maturation of the complex postsynaptic tectal neuron dendritic arbors (2), regulates the maturation and stabilization of retinotectal synapses (3), refines the microcircuitry within the tectum (4), and sets the level of intrinsic excitability expressed by tectal neurons (5).

Meanwhile, back in the water, the visual systems of amphibians and fish are developing, forming topographic maps, refining receptive fields, and building circuits to detect and process visual information—all in the absence of spontaneous retinal waves (Figure 2). Visual responses can be observed in Xenopus tadpoles as soon as RGC axons reach the optic tectum and begin forming synapses onto dendrites of tectal neurons, which happens at developmental stage 39/40, only 4–5 days postfertilization (dpf; Holt and Harris, 1983). Below, we review several consequences of visually driven activity on the development and function of the immature retinotectal circuit.

### Development and Refinement of The Topographic Retinotectal Map

One consequence of visually-driven activity is the development and refinement of a retinotopic projection in the optic tectal neuropil. Studies by Holt and Harris (1983) indicate that the first visual responses that can be recorded in the optic tectum already have a crude spatial organization, suggesting that retinal axons may form a rough topographic map as soon as retinal afferents innervate tectal neurons. Experiments, largely in chicks and amniotes, provided evidence that gradients of cell surface ligands and receptors located on RGCs and central retinal targets guide retinal axons to topographically matched target locations (reviewed in McLaughlin et al., 2003; Feldheim and O’Leary, 2010), and recent work showed that similar gradients are present in frog and tadpole optic tecta (Higenell et al., 2012). Reh and Constantine-Paton (1984) published two landmark articles: one demonstrated that individual RGC axon arbors shift their positions within the optic tectum as the retina and optic tectum enlarge during development to maintain a refined retinotopic projection. The second showed that blocking action potential activity traveling from the retina to the tectum disorganized the retinotectal projection (Reh and Constantine-Paton, 1985). Even before the discovery of spontaneous retinal waves (Meister et al., 1991), studies showing that activity in neighboring RGCs was highly correlated (Mastronarde, 1989) together with the studies from Reh and Constantine-Paton showing that blocking action potential activity from the retina to targets, provided critical support for the idea that patterned retinal input instructed the development of topographic visual projections by regulating the termination site of axons in the target. Subsequent work indicated that tectal N-Methyl-D-aspartate (NMDA) receptor activity is required for the development and maintenance of organized retinotectal projections (Cline et al., 1987; Cline and Constantine-Paton, 1989; Ruthazer et al., 2003). Synthesizing this body of work with work from other systems led to the idea that retinal input, be it from natural visual input in anamniotes or from spontaneous retinal waves in amniotes, instructs the development of organized visual projections (Udin and Fawcett, 1988; Constantine-Paton et al., 1990).

A core element of this conceptual framework is that correlated activity in neighboring afferents is detected by postsynaptic NMDA receptors based on principles of Hebbian plasticity models and spike timing dependent plasticity (STDP), suggesting that STDP-based mechanisms might refine the topographic map. Cellular mechanisms underlying topographic map refinement can be evaluated by examining dynamic rearrangements of retinotectal axon arbors *in vivo* (Ruthazer et al., 2003; Munz et al., 2014). These types of studies have been instrumental in identifying rules by which correlated activity governs axon remodeling underlying topographic map plasticity, as reviewed by Kutsarova et al. (2016). Topographic map refinement can also be read out as a refinement of the size of visual receptive fields in tectal neurons, and this refinement is thought to occur by engaging long-term potentiation and depression synaptic plasticity mechanisms (Ruthazer and Aizenman, 2010). Several experiments lay the groundwork for this important cross-cutting concept. In the first *in vivo* demonstration of STDP, Zhang et al. (1998) used a stimulation electrode to activate RGCs and postsynaptic recordings in tectal neurons, to show that activation of presynaptic RGC inputs could induce either LTP or LTD, depending on the timing of the incoming RGC action potential relative to the depolarization of the postsynaptic tectal neuron. Furthermore, a repetitive dimming light stimulus to the eye also induced LTP of retinotectal synapses in the contralateral tectum (Zhang et al., 2000).

Between stages 44 and 49, experience-dependent refinement of the retinotectal projection decreases receptive field size (Tao and Poo, 2005; Dong et al., 2009). This may occur by STDP-based mechanisms (Tao et al., 2001), although STDP of retinotectal synapses cannot be induced throughout this developmental period (Tsui et al., 2010), suggesting that other mechanisms contribute to receptive field and topographic map refinement (Ruthazer and Aizenman, 2010). Indeed, brief training with visual experience induces transcriptional and translational changes that affect visual responses and visually evoked behaviors (Dong et al., 2009; Schwartz et al., 2009, 2011; Shen et al., 2014), suggesting that further exploration will reveal additional cellular and molecular mechanisms regulating topographic map refinement and the development of neuronal response properties. As discussed in more detail below, refinement of visual receptive fields and the topographic map is necessary for tadpoles’ visually guided avoidance responses.

The receptive fields of tadpole tectal neurons display robust forms of activity-dependent plasticity. At developmental stage 45, tectal neurons are not direction selective, meaning that they respond equally to all moving stimuli, regardless of the direction of movement of the visual stimulus. However, training tectal neurons by projecting unidirectional moving bars of light onto the retina induces direction selectivity in tectal neurons for the trained direction (Engert et al., 2002). In other words, after unidirectional training, neurons respond best to the trained direction. It is interesting to note that this training-induced directional selectivity involved an asymmetric shift in the neuron’s receptive field, with new responsiveness to earlier-activated bar locations (Engert et al., 2002) suggestive of a STDP-type of plasticity in action.

**Figure 3:**
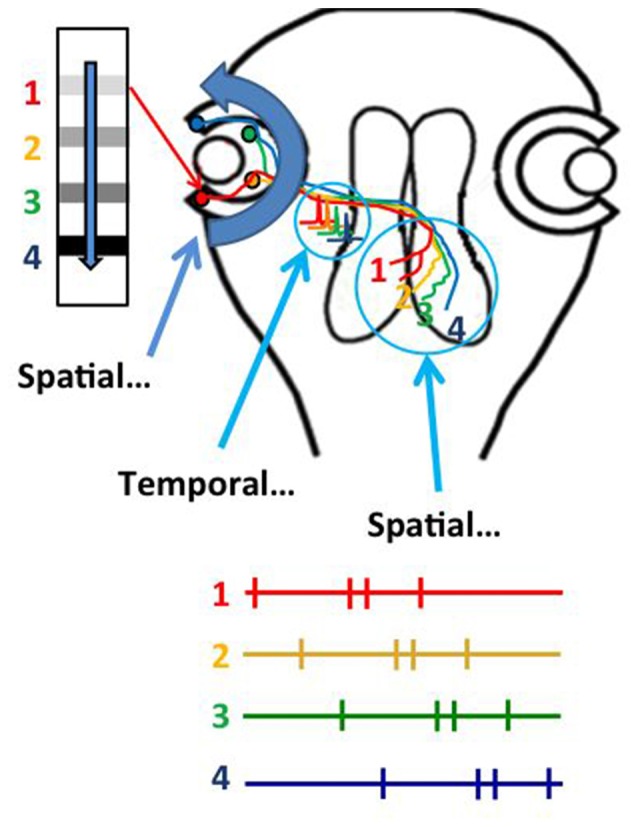
**Experience-dependent refinement of the retinotectal map occurs by a spatial to temporal to spatial (STS) transformation of visual information.** Natural anterior to posterior motion of objects in the visual scene, or optic flow, activates RGCs from a temporal to nasal sequence, transforming spatial information in the visual scene into temporal information in the sequence of action potential activity in RGCs. The temporal information in firing sequence is transformed into an ordered spatial distribution of RGC axons in the tectal neuropil, based on a rule in which axons that fire first occupy more rostral positions within the tectal target. The STS mechanism ensures that retinal inputs map topographically across the entire target area.

Several studies indicate that STDP mechanisms distort visual receptive field properties and topographic projections of sensory input (Engert et al., 2002; Fu et al., 2002; Sundberg et al., 2006; Lim et al., 2010). The fact that receptive field properties and topographic maps are relatively stable suggests that mechanisms other than STDP might function to limit receptive field and map distortion. Indeed, a recent study demonstrates that the natural visual experience in response to optic flow from the constant forward swimming motion of tadpoles instructs the refinement of the retinotectal topographic map (Hiramoto and Cline, 2014). We noticed, as you could too, that tadpoles always swim forward, producing a constant source of anterior to posterior visual stimulation in the retina. This would produce a constant sequence of RGC activity from temporal to nasal retina. Rearing animals for 4 days under conditions in which the only visual experience they received was anterior to posterior moving bar stimulus resulted in the development of a refined retinotopic projection, whereas rearing animals with posterior to anterior moving bar stimulus prevented the refinement of the retinotectal projection. Temporal RGCs terminate in rostral tectum and RGCs in incrementally more nasal positions along the temporal-nasal axis terminate in correspondingly more caudal positions in the optic tectum (Figure 3). This suggested that the sequence of activity in temporal to nasal RGCs in response to the anterior to posterior moving stimuli might organize the RGC axons along the rostrocaudal axis of the tectum. Further analysis indicated that the axons of RGCs that were active earlier than converging RGC axons would shift their positions to more rostral tectal locations and that RGC axons that were active later than converging inputs would shift to more caudal tectal locations. The *in vivo* imaging protocols used in this study even provided the spatial and temporal resolution to show that the positions of individual axons could be arbitrarily shifted along the rostrocaudal tectal axis by changing the relative sequence of activity in the RGCs. Overall, this study supports a model in which the spatial location of objects in the visual field is encoded in the temporal sequence of RGC activity as the objects move in an anterior to posterior direction across the retina, and that this temporal sequence of RGC activity is then transformed into the spatial arrangement of RGC axon arbors within the target optic tectum. The spatial to temporal to spatial (STS) transformation of information operates throughout the temporal to nasal axis of the retina and the rostral to caudal axis of the tectum, suggesting that one critical function of the STS mechanism may be to calibrate sensory information from the periphery to the target area devoted to that sensory projection. Importantly, this mechanism would this explain how sensory maps are customized to each individual, and accommodate individual differences in physical dimensions or positions of the sensory periphery. In addition, this mechanism may also underlie plasticity of topographic projections in response to changes in the sensory periphery or central targets (Garraghty and Kaas, 1992), as shown for instance with retinal scotomas (Gilbert, 1992; Gilbert and Wiesel, 1992), loss of digits or limbs, or stroke (Nudo and Friel, 1999), as well as classic studies on retinotectal map plasticity in which removal of half a retina or half the tectum results in expansion and compression, respectively, of the retinotopic projections (Udin and Fawcett, 1988). STDP and STS likely operate in concert, with STDP-based mechanisms allowing critical rapid modifications in neuronal response properties and STS maintaining a scaled topographic projection across the available target space.

The STS mechanism likely operates in amniotes as well as anamniotes, but in amniotes it is the temporal to nasal direction of spontaneous waves (Stafford et al., 2009; Ackman et al., 2012), rather than anterior to posterior motion of natural optic flow, that organizes the rostrocaudal mapping of retinal afferents in the SC, as well as the topographic projections in higher order visual centers that are likely organized by propagating spontaneous waves originating in the retina (Stafford et al., 2009; Ackman et al., 2012; Ackman and Crair, 2014; Burbridge et al., 2014).

### Structural and Functional Development of Tectal Neurons

A second consequence of sensory experience is the effect on the structural and functional development of tectal neurons and their connectivity in nascent circuits. Although the majority of experimental work on this topic in *Xenopus* has been done by manipulating visual inputs to the tectum, mechanosensory experience, which enters the tectum from the hindbrain (Deeg et al., 2009; Hiramoto and Cline, 2009), is also likely to play a significant role in governing the development of tectal cell structure, function and connectivity. Because of their transparency at early developmental stages, their external development, and the ease with which *in vivo* time-lapse imaging, electrophysiology and gene manipulation can be accomplished, *Xenopus* tadpoles have been a particularly valuable experimental system in which to investigate neuronal development in intact developing animals.

Single cell labeling of optic tectal cells followed by *in vivo* time-lapse imaging showed that tectal neuron dendrites go through a rapid phase of growth, lasting several days, followed by a plateau in growth rate (Wu et al., 1999; Cline, 2001). Although one could imagine that dendritic arbor growth occurs by lengthening pre-existing branches and adding new branches, collecting *in vivo* time-lapse images at relatively short intervals, such as every 10–30 min over several hours, indicated that dendritic arbor growth occurs by dynamic addition and retraction of branches. Furthermore, net growth or net retraction of the entire arbor structure occurs as a result of relatively more branch additions and extensions than branch retractions, or conversely more retractions than additions, respectively (Dailey and Smith, 1996; Rajan and Cline, 1998; Haas et al., 2006; Cline and Haas, 2008; Ewald et al., 2008). It is interesting to note that the branch dynamics underlying arbor growth persist in mature neurons when the arbor structure is stable, albeit at a slower rate (Wong and Ghosh, 2002; Lee et al., 2006, 2008; He et al., 2016), suggesting that mechanisms that regulate developmental dendritic dynamics also regulate dendritic structural plasticity in mature neurons.

During the initial period of dendrite elaboration, analysis of individual neurons showed considerable spatial and temporal heterogeneity in dendritic arbor growth patterns. The developing dendritic arbors in some neurons would show a rapid spurt of growth and then remain stable for a period before they resumed growth. Others elaborated one region of their dendritic arbor at the same time that other regions remained stable or were retracted (Rajan and Cline, 1998; Wu and Cline, 1998, 2003; Rajan et al., 1999; Wu et al., 1999). Optic tectal neurons receive glutamatergic and GABAergic synaptic input even as they elaborate their dendritic arbors (Wu et al., 1996; Akerman and Cline, 2006). We suspected that the spatial and temporal growth heterogeneity might be readouts of activity-dependent signaling that affected branch dynamics. Indeed this idea was supported by experiments showing that blocking NMDA receptors decreased dendritic arbor growth by altering branch dynamics in newly differentiating tectal neurons (Rajan and Cline, 1998; Rajan et al., 1999). By contrast, blocking α-amino-3-hydroxy-5-methyl-4-isoxazolepropionic acid (AMPA) receptors or action potential activity in these relatively immature neurons had no significant effect (Rajan and Cline, 1998). These observations suggested that the glutamatergic retinal inputs might be regulating dendritic arbor growth of postsynaptic tectal neurons by regulating branch dynamics via signaling through synapses. Electrophysiological recordings from tectal neurons demonstrated that relatively immature tectal neurons respond to retinal axon stimulation and that transmission at their glutamatergic synapses was predominantly mediated by NMDA receptor conductances and that AMPA receptors were trafficked into synapses by a Calcium/calmodulin-dependent protein kinase II (CaMKII)-dependent mechanism (Wu et al., 1996; Wu and Cline, 1998). As neurons mature, their dendritic arbors become more complex and transmission at their glutamatergic synapses becomes stronger through the addition of AMPA receptors. Consistent with this synaptic maturation profile, pharmacologically blocking AMPA receptors selectively interferes with dendritic arbor elaboration in more mature neurons (Rajan and Cline, 1998). Furthermore, interfering with AMPA receptor trafficking, by manipulating CaMKII function (Wu et al., 1996; Wu and Cline, 1998), or by expressing a peptide corresponding to the C-terminal of GluA subunits, called CTP, drastically altered dendritic arbor growth (Haas et al., 2006).

Experiments in which single tectal neurons were imaged in intact animals before and after brief 4 h periods during which animals were either kept in the dark or exposed to a motion stimulus, provided direct demonstration of the role of visual experience on dendritic arbor growth (Sin et al., 2002). Dendritic arbor growth rates were relatively low over the 4 h period in the dark and increased significantly over the 4 h period with visual stimulation. This imaging protocol allows comparison of growth rates over time in individual neurons with and without visual stimulation and therefore provides greater power to detect experience-dependent changes in structural plasticity and to identify cellular and molecular mechanisms regulating experience-dependent dendritic arbor development.

**Figure 4:**
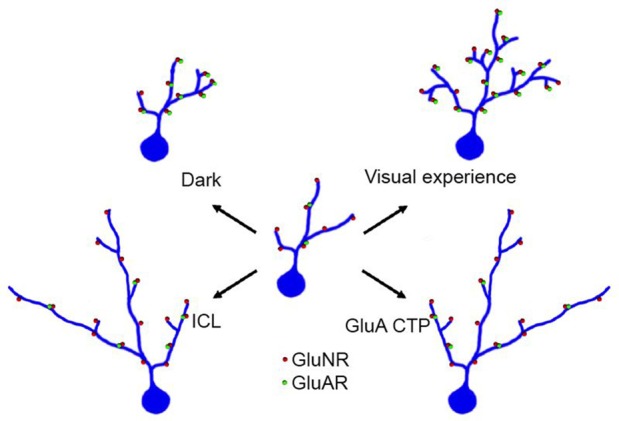
**Visual experience enhances dendritic arbor development through effects on excitatory and inhibitory synaptic transmission.** An immature optic tectal neuron is schematized in the center of the figure and structural changes in dendritic arbor in response to different conditions are shown radiating from the center. Under conditions in which tadpoles receive visual stimulation, optic tectal neurons elaboration complex dendritic arbors and their excitatory synapses mature and increase in strength by increasing the ratio of AMPA/NMDA type glutamate receptors. Raising tadpoles in the dark decreases elaboration of the dendritic arbor. Expressing GluA C terminal peptides (GluA CTP) impairs AMPA receptor trafficking, decreases excitatory synapses and decreases complexity of dendritic arbors. Expressing intracellular loop (ICL), which impairs GABA_A_R residence at synapses and decreases inhibitory synaptic inputs onto tectal neurons, also decreases complexity of dendritic arbors.

A core element of mechanisms underlying experience-dependent dendritic arbor development is the regulation of glutamate receptor trafficking. Visual experience increased the strength of retinotectal glutamatergic synapses by increasing the contribution of AMPA receptors, or the AMPA/NMDA ratio, at synapses (Engert et al., 2002; Haas et al., 2006; Shen et al., 2011). Blocking AMPA receptor trafficking by expression of CTP blocks visual experience-dependent dendritic arbor growth (Figure 4; Haas et al., 2006). This in turn predicts that manipulating synaptic proteins that affect AMPA receptor trafficking will also affect dendritic arbor elaboration and specifically experience-dependent dendritic arbor elaboration. This prediction has been borne out in recent studies of the transmembrane AMPA receptor regulatory proteins (TARPS), a family of proteins, which regulate AMPA receptor trafficking and modulate their function (Chen et al., 2000). A subset of type I TARPs enhance AMPA receptor trafficking to postsynaptic sites and also regulate activity-dependent dendritic arbor development in cortical pyramidal neurons (Hamad et al., 2014). Conversely, CPG15, aka neuritin, an activity-induced growth factor (Nedivi et al., 1996; Fujino et al., 2003; Harwell et al., 2005; Javaherian and Cline, 2005) increases AMPA receptor trafficking into retinotectal synapses and dramatically increases dendritic arbor elaboration and retrogradely increases elaboration of presynaptic retinal axon arbors (Nedivi et al., 1998; Cantallops et al., 2000).

Other postsynaptic density proteins, including ion channels, cell adhesion molecules, cytosolic signaling proteins, cytoskeletal proteins and scaffolding proteins (Kim and Sheng, 2004; Sheng and Hoogenraad, 2007) may affect NMDA or AMPA receptor mediated synaptic transmission and thereby affect experience-dependent dendritic arbor growth. This generalization is important because it suggests that mechanistic understanding of the assembly, function and stability of synapses will in turn identify mechanisms that affect dendritic arbor development and circuit connectivity in the developing brain. In particular, a significant number of postsynaptic density proteins are candidate disease genes for neurodevelopmental disorders such as autism and schizophrenia (Ebrahimi-Fakhari and Sahin, 2015), supporting the idea of neurodevelopment origins of complex neurological diseases that manifest at later life stages.

It is interesting that activity-dependent mechanisms can also restrict dendritic arbor growth. For instance, electrophysiological experiments of synaptic maturation in *Xenopus* tectal optic neurons indicate that increased α-CaMKII activity both increases glutamatergic synaptic strength (Wu et al., 1996) and stabilized dendritic arbor structure by reducing rates of branch additions and retractions (Wu and Cline, 1998). α-CaMKII, a multifunctional calcium and calmodulin-dependent kinase, acts downstream of synaptic activity-dependent increases in calcium to regulate synaptic strength (Lisman et al., 2002) and cytoskeletal dynamics (McVicker et al., 2015), for instance via GTPases (Sin et al., 2002; Ghiretti et al., 2014). Mechanisms limiting neuronal arbor size are less well studied than those that enhance arbor growth, but are under active investigation as reviewed in Koleske (2013).

Although studies of sensory experience-dependent development have focused research on excitatory synaptic input mediated effects on dendrite development, inhibitory synaptic activity driven by sensory input also regulates dendritic arbor development. The roles of inhibitory GABAergic or glycinergic synaptic transmission in regulating dendritic arbor development depends on the expression of chloride transporters and therefore whether the transmitter depolarizes or hyperpolarizes the postsynaptic neuron. Activation of ionotropic type A GABA receptors (GABA_A_R) in young neurons increases process outgrowth and synaptogenesis, possibly mediated by GABA—induced excitation (Barbin et al., 1993; Ben-Ari, 2002; Cancedda et al., 2007). Blocking inhibitory GABAergic transmission in preparations containing mature neurons increases process outgrowth (Wayman et al., 2006) by increasing activity indirectly. Similarly, glycinergic transmission affects dendritic arbor development, both at early stages of development, when it is depolarizing (Maric et al., 2001; Tapia et al., 2001), and later, when glycinergic transmission is inhibitory (Sanes and Chokshi, 1992; Sanes et al., 1992; Sanes and Hafidi, 1996). Blocking glycinergic input with strychnine increased dendritic arbor size, suggesting that the normal function of inhibitory input is to restrain dendrite growth. Although these experiments indicate that inhibitory transmission affects dendritic arbor development, the experiments produce circuit-wide effects on activity levels that confound the interpretation of changes in neuronal structure (Ben-Ari et al., 1989; Chen et al., 1996; Tapia et al., 2001).

One useful strategy to study the effects of inhibitory synaptic input on neuronal development and function is to express a peptide corresponding to the intracellular loop of γ2 subunit of GABA_A_R, called intracellular loop (ICL), which prevents γ2 subunit—containing GABA_A_R from anchoring at synapses (Alldred et al., 2005; Christie et al., 2006) and allows cell autonomous manipulations of inhibitory input. Electrophysiological experiments show that ICL decreased inhibitory synaptic inputs in neurons that expressed ICL but not in untransfected neurons or those expressing a mutant ICL, called mICl, and furthermore that ICL increased the ratio of excitatory to inhibitory synaptic activity in ICL-expressing neurons. Time-lapse 2 photon images of optic tectal neurons *in vivo* collected at daily intervals showed that ICL-expressing neurons have less elaborate dendritic arbors that span a larger area of the tectal neuropil compared to controls. Images collected at shorter intervals indicated that the decrease in arbor branches arose from a decrease in the numbers of new branch additions to the arbors (Shen et al., 2009), rather than in increase in branch retractions as seen when AMPA receptor trafficking into synapses was disrupted (Haas et al., 2006). Decreasing inhibitory input, which likely increased the balance of excitation to inhibition, blocked the visual-experience dependent increase in dendritic arbor complexity. These results suggest that a change in the balance of excitatory to inhibitory inputs disrupts dendritic arbor development. Given the current evidence that the balance of excitation to inhibition is critical for normal brain function, and that neurodevelopmental disorders, such as autism spectrum disorders disrupt the balance of excitation to inhibition (Gatto and Broadie, 2010; Paluszkiewicz et al., 2011; Calfa et al., 2015), it will be of great interest to determine how changes in the relative balance of excitatory to inhibitory synaptic inputs affect signaling pathways and cellular machinery that regulate dendritic arbor development.

### Development and Maturation of Local Tectal Circuitry

A third effect of visual experience on the development of the visual system in tadpoles is the maturation of local tectal microcircuitry. In addition to direct activation of tectal neurons, visually driven RGC input also activates local recurrent microcircuitry within the tectum (Pratt et al., 2008; Xu et al., 2011). Relatively long lasting, and capable of eliciting the firing of multiple action potentials in a given tectal neuron, this recurrent activity adds a temporal dimension to the visual response. Although the exact function of the polysynaptic recurrent activity is not completely understood, it likely codes for different aspects of the visual stimuli and/or response, similar to the recurrent activity in the SC (Sparks, 1986; Moschovakis et al., 2001). Another possibility is that recurrent activity maintains neurons at relatively depolarized potentials and thereby boosts their ability to respond to incoming input (Haider et al., 2007). Like the monosynaptic response, the local polysynaptic activity undergoes activity-dependent refinement between stages 44 and 49. Refinement of the local microcircuitry is characterized by visually-evoked responses becoming more compressed and occurring closer in time to the preceding monosynaptic response (Pratt et al., 2008). Dampening RGC input by blocking both NMDA and calcium-permeable AMPA receptors during this time resulted in responses that were similar to those seen in stage 44 circuits, suggesting that retinal input contributes to the maturation of microcircuitry (Pratt et al., 2008). It is interesting to note that visual experience-dependent maturation of temporal response properties in local tectal circuitry occurs by STDP rules. This was shown in an isolated brain preparation and in intact tadpoles. In the isolated brain preparation, pairs of stimuli were delivered to the retinal inputs, so that the second stimulus was timed to occur in the midst of the recurrent portion of the response activated by the first stimulus. This stimulus condition shifted the temporal properties of the recurrent activity in accordance with STDP rules. When tadpoles were exposed to pairs of visual stimuli with different interstimulus intervals for 4 h, the temporal properties of recurrent tectal activity were also shifted, as seen in the *ex vivo* brain preparation. This important observation indicated that the visual system connections and therefore visual system responses in intact animals are “trained” to respond optimally to the temporal properties of predominant stimuli.

The spatial pattern of connectivity of optic tectum microcircuits is also affected by visual experience. This was demonstrated by bulk-loading tectal neurons with calcium indicators so that calcium transients could be imaged simultaneously in a large population of neurons. Activating RGC inputs with a whole field light on stimulus to the retina demonstrated that the degree of correlated activity across the tectum significantly increased between developmental stages 44 and 49. Furthermore, this increased spatial correlation depends on visual experience, as it is almost completely eliminated by dark rearing (Xu et al., 2011). These experiments indicate that the development of both the spatial and temporal features of tectal circuit responses are experience-dependent.

Synaptic input from the retina can also regulate a tectal neuron’s intrinsic excitability—the ease in which a neuron fires action potentials. Because recurrent activity is generated by local tectal-tectal connections, the intrinsic excitability of the individual tectal neurons greatly impacts the strength and pattern of this local activity (Dong and Aizenman, 2012). Furthermore, the long range projections of tectal neurons provides afferent input to the brainstem, which is then relayed ultimately to spinal cord circuits to elicit a swimming response (Khakhalin et al., 2014). Therefore, changes in intrinsic excitability would be expected to impact both the local tectal microcircuitry, as well as the downstream target circuits. Between developmental stages 45 and 49, the number of synapses, and so the overall amount of synaptic drive received by tectal neurons, increases dramatically (Pratt and Aizenman, 2007). The developmental increase in synaptic drive received by tectal neurons triggers a compensatory response in their intrinsic excitability. In other words, as synaptic drive increases, intrinsic excitability decreases. Importantly, dampening the increase in synaptic drive by expressing a truncated AMPA receptor subunit prevented the decrease in intrinsic excitability. In fact decreasing synaptic input causes a significant increase in intrinsic excitability, illustrating that intrinsic excitability adjusts bidirectionally in response to changes in synaptic drive, and not the other way around (Pratt and Aizenman, 2007). Similarly, 4 h of enhanced visual experience induces a decrease in synaptic drive by activating polyamine blockade of current through AMPA ion channels—a protective mechanism in times of synaptic over-activation (Bell et al., 2011). This downregulation of synaptic drive increases intrinsic excitability, which overall, is thought to increase the signal to noise ratio (Aizenman et al., 2003).

Perhaps the most enchanting demonstration of the effect of visual experience on tectal neuron action potential firing is a study by van Rheede et al. (2015). First, the authors establish that at early larval stages 42–44, the time in development when RGC input has just started to form nascent synapses onto tectal neurons, a large fraction of tectal neurons do not fire action potentials in response to a “light-off” stimulus projected onto the retina. Interestingly, these neurons can fire action potentials in response to current injection, but they don’t fire in response to visually-driven input. The non-spiking neurons can be converted to spiking neurons with 15 min of visual conditioning, consisting of a drifting bar of light (van Rheede et al., 2015). Better yet, non-spiking neurons can be converted to spiking neurons by showing the tadpole underwater scenes from the documentary “*Planet Earth*” (BBC). When tadpoles are shown a black scene, non-spiking neurons are not converted to spiking ones. The mechanism underlying the conversion to spiking involves changes in synaptic strength, while no changes in intrinsic excitability were detected. Overall, during development of the retinotectal circuit, the input provided by RGC activation shapes the functional development of tectal microcircuitry, making it more consistent and faster.

## Conclusion

Visual experience plays a critical function in the development and maturation of the visual circuitry in anamniotes, including the development of the topographic retinotectal projection, retinotectal synaptic properties, tectal neuronal morphological development, as well as broader properties of tectal circuitry including connectivity underlying recurrent activity. Together, these studies provide strong evidence that sensory input drives the development and maturation of diverse synaptic, neuronal and circuit properties. These events likely require changes in gene expression and translation. Although, we did not review studies on activity-induced gene transcription or translation, hundreds of transcripts are known to be regulated by activity (Nedivi et al., 1993; Loebrich and Nedivi, 2009), and analysis of many activity-regulated transcripts has shown they affect nervous system development (Loebrich and Nedivi, 2009). Similarly, protein translation can also be regulated by activity, with an effect on visual system plasticity (Shen et al., 2014). Given the functional parallel between spontaneous waves of activity in amniotes and the role of visual experience in visual system development in non-amniotes, it seems likely that spontaneous waves of activity propagated from the retina throughout the visual system (Ackman and Crair, 2014) will have widespread repercussions for visual system development in these systems. Finally, recent studies have demonstrated striking parallels in activity-dependent cellular and molecular mechanisms governing synaptic and circuit maturation in non-sensory brain circuits (Kozorovitskiy et al., 2012) as we have reviewed above, suggesting that the fundamental mechanistic principles of brain circuit development identified in the developing *Xenopus* visual system are evolutionarily conserved and apply broadly to brain circuit development across phyla and brain regions.

## Author Contributions

KGP, MH and HTC discussed the concept. KGP and HTC co-wrote the article.

## Funding

KGP is supported by NIH P30-GM-32128. HTC is supported by NIH EY11261 and an endowment from the Hahn Family Foundation.

## Conflict of Interest Statement

The authors declare that the research was conducted in the absence of any commercial or financial relationships that could be construed as a potential conflict of interest.
